# 1262. The Impact of Antimicrobial Stewardship on Antimicrobial Consumption and Resistance Trends at a Tertiary Care Center in Beirut

**DOI:** 10.1093/ofid/ofad500.1102

**Published:** 2023-11-27

**Authors:** Nisrine Haddad, Tamara Abdallah, Nesrine Rizk, Rony Zeenny, Souha S Kanj

**Affiliations:** American University of Beirut Medical Center, Beirut, Beyrouth, Lebanon; American University of Beirut Medical Center, Beirut, Beyrouth, Lebanon; American University of Beirut, Beirut, Beyrouth, Lebanon; American University of Beirut medical Center, Beirut, Beyrouth, Lebanon; American University of Beirut Medical Center, Beirut, Beyrouth, Lebanon

## Abstract

**Background:**

Antimicrobial Resistance (AMR) is a threat to public health, causing increased morbidity and mortality particularly among hospitalized patients. The COVID-19 pandemic has further worsened the situation globally, causing a surge in the number of critically ill patients, and an increase in antimicrobial consumption resulting in the spread of infections with multidrug-resistant organisms (MDROs). Antimicrobial Stewardship Programs (ASP) play a crucial role in optimizing antimicrobial consumption to fight AMR. In this study, we evaluate the impact of ASP interventions on antibiotic consumption and trends of AMR among bacterial pathogens at a tertiary care center in Beirut before and after the pandemic.

**Methods:**

ASP team recommendations are labeled as interventions. We compiled the ASP interventions from January 2019 until December 2021. Data on antimicrobial consumption, expressed as defined daily dose (DDD) per 100 patient days, for all anti-infectives and restricted anti-infectives was collected per quarter. Data on MDRO resistance trends were obtained from the clinical microbiology laboratory from 2019 to 2022.

**Results:**

The number of ASP recommendations is reported in Figure 1. There is a noticeable correlation between COVID-19 surges in Lebanon and the number of ASP interventions. We note an increase in all antimicrobial consumption after the onset of the pandemic, with a peak in Q4 2020 (142.8 DDD/100 patient days) (Figure 2), and in Q1 2021 for restricted antimicrobials (79.1 DDD/100 patient days) (Figure 3). Importantly, there were no noteworthy changes in resistance patterns for MDR gram-negative organisms, except for MDR *Acinetobacter baumannii* and Carbapenem resistant *Klebsiella* spp., which decreased by 27% and 8%, respectively. We witnessed a rise in Gram-positive resistance trends for Methicillin-resistant *Staphylococcus aureus* and Vancomycin-resistant *Enterococcus* (f29% to 33% and 9% to 14% in 2019 to 2022, respectively).

**Figure 1.**
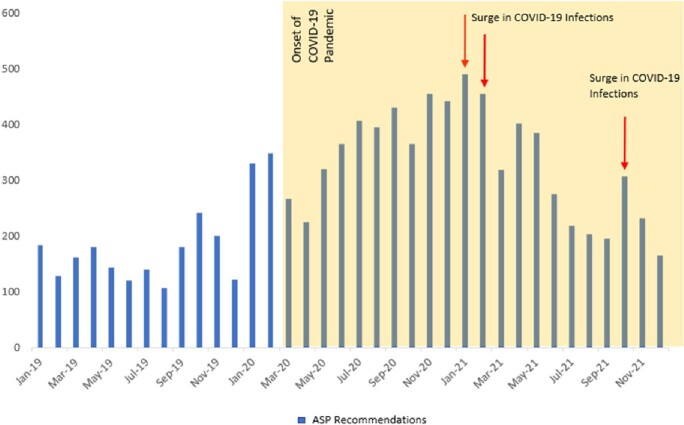
Number of ASP Interventions Pre- and During COVID-19

**Figure 2.**
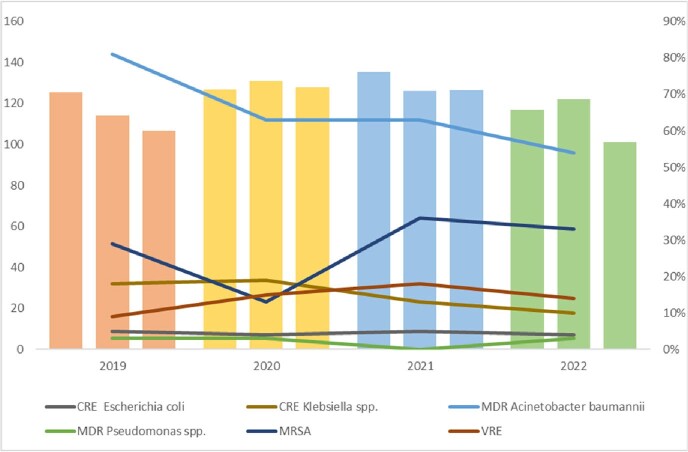
All Antimicrobials Defined Daily Dose per 100 Patient Days and Resistance Trends of MDROs

**Figure 3.**
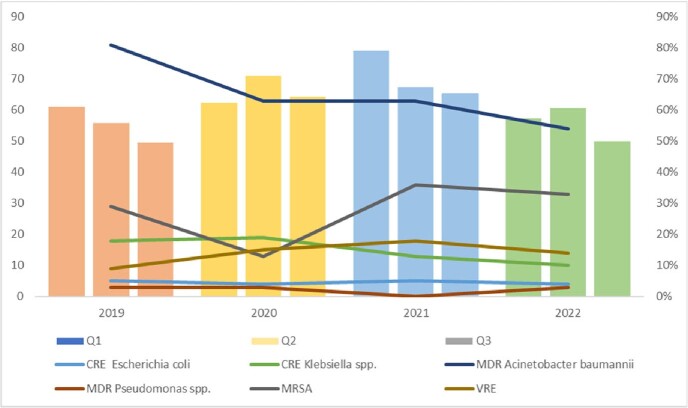
Restricted Antimicrobials Defined Daily Dose per 100 Patient Days and Resistance Trends of MDROs

**Conclusion:**

This study highlights the importance of ASP activities despite and during a pandemic. The results emphasize the role of ASP to mitigate the impact of the pandemic on antimicrobial resistance. The ASP team maintained its operations, monitoring antibiotic consumption and providing recommendations to limit antibiotic misuse.

**Disclosures:**

**Souha S Kanj, MD**, Gilead: Advisor/Consultant|Menarini: Advisor/Consultant|MSD: Advisor/Consultant|Pfizer: Advisor/Consultant

